# Porphyrin–phospholipid liposomes permeabilized by near-infrared light

**DOI:** 10.1038/ncomms4546

**Published:** 2014-04-03

**Authors:** Kevin A. Carter, Shuai Shao, Matthew I. Hoopes, Dandan Luo, Bilal Ahsan, Vladimir M. Grigoryants, Wentao Song, Haoyuan Huang, Guojian Zhang, Ravindra K. Pandey, Jumin Geng, Blaine A. Pfeifer, Charles P. Scholes, Joaquin Ortega, Mikko Karttunen, Jonathan F. Lovell

**Affiliations:** 1 Department of Biomedical Engineering, University at Buffalo, State University of New York, Buffalo, New York 14260, USA; 2 Department of Chemical and Biological Engineering, University at Buffalo, State University of New York, Buffalo, New York 14260, USA; 3 Department of Chemistry and Waterloo Institute for Nanotechnology, University of Waterloo, Waterloo, Ontario, Canada N2L 3G1; 4 Department of Biochemistry and Biomedical Sciences and M. G. DeGroote Institute for Infectious Diseases Research, McMaster University, Hamilton, Ontario, Canada L8S4L8; 5 Department of Chemistry, University at Albany, State University of New York, Albany, New York 12222, USA; 6 PDT Center, Roswell Park Cancer Institute, Buffalo, New York 14263, USA

## Abstract

The delivery of therapeutic compounds to target tissues is a central challenge in treating disease. Externally controlled drug release systems hold potential to selectively enhance localized delivery. Here we describe liposomes doped with porphyrin–phospholipid that are permeabilized directly by near-infrared light. Molecular dynamics simulations identified a novel light-absorbing monomer esterified from clinically approved components predicted and experimentally demonstrated to give rise to a more stable porphyrin bilayer. Light-induced membrane permeabilization is enabled with liposomal inclusion of 10 molar % porphyrin–phospholipid and occurs in the absence of bulk or nanoscale heating. Liposomes reseal following laser exposure and permeability is modulated by varying porphyrin–phospholipid doping, irradiation intensity or irradiation duration. Porphyrin–phospholipid liposomes demonstrate spatial control of release of entrapped gentamicin and temporal control of release of entrapped fluorophores following intratumoral injection. Following systemic administration, laser irradiation enhances deposition of actively loaded doxorubicin in mouse xenografts, enabling an effective single-treatment antitumour therapy.

## Abstract (web summary)

The delivery of therapeutics using an external trigger is an attractive route for the improvement of targeted disease treatment. Here, the authors have discovered a porphyrin–phospholipid liposome for light-controlled membrane permeabilization and use the system to deliver an anticancer drug *in vivo*.

## Graphical abstract

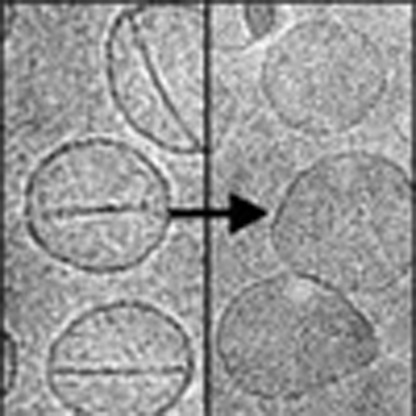

Several clinically approved nanocarriers such as liposomes have been developed to improve the biodistribution and anticancer efficacy of various drugs^1^,^2^. However, delivery is hampered by physiological barriers and release kinetics so that biodistribution and bioavailability are almost inevitably suboptimal^3^,^4^. To address this problem, numerous diverse strategies have been pursued that make use of external stimuli to trigger local drug release^5^,^6^,^7^,^8^,^9^. Over 30 years ago, it was discovered that dipalmitoyl phosphatidylcholine liposomes, which have a phase-transition temperature near 41 °C, release entrapped drugs when heated to 44 °C (ref. 10). Subsequently, this formulation was optimized by including a small portion of single side-chain phospholipids, leading to the development of lysolipid-containing temperature-sensitive liposomes, which have progressed to human clinical trials^11^,^12^,^13^. Liposomal release based on heating to lipid phase-transition temperatures has been expanded to other thermal transduction methods including ultrasonic tissue heating^14^, magnetic field heating using magnetoliposomes^15^,^16^ and near-infrared (NIR) photothermal heating via gold coating^17^,^18^, tethering^19^ and co-administration^20^. Analogously, NIR photothermal release from cargo-loaded gold nanoparticles has been demonstrated for adsorbed and entrapped drugs^21^ and gold nanocages coated with polymers that exhibit phase transitions at 39 °C (ref. 22). Other light-triggered release mechanisms have been proposed that make use of ultraviolet light to induce chemical reactions, although adaptation to biological systems is impractical because of its reactivity and poor penetration^23^. Overall, it has proved challenging to develop a nanocarrier that in physiological conditions can stably retain cargo in the absence of an external stimulus but release it in its presence. As nearly all biocompatible triggered release mechanisms described so far are based on a thermal transition a few degrees above body temperature, these carriers naturally exhibit a substantial amount of background release at 37 °C (ref. 24). Using temperature-triggered nanocarriers with phase transitions at even higher temperatures is not practical, as the elevated heating required for release would in itself destroy the target tissue and could induce stasis within tumour vesicles that could impede drug delivery^25^. Even for current methods that rely on target tissue heating to a few degrees above body temperature, heat activation alone can induce apoptosis^26^ and has a drastic impact on drug delivery^24^ that confounds the effects of drug release and the triggering stimuli.

Here we introduce a fundamentally new method for triggered drug release that makes use of porphyrin–phospholipid (PoP)-doped liposomes that overcomes some of the limitations of previously described systems. Molecular dynamics (MD) simulations are used to identify a stable new porphyrin–lipid monomer that gives rise to more stable bilayers. At appropriate PoP-doping levels (~10 molar (mol.) %), PoP-liposomes can be permeabilized by irradiation with NIR light. PoP-liposomes can be loaded with a range of cargos including fluorophores, antibiotics and chemotherapeutics, and release these on demand with excellent spatial and temporal control.

## Results

### MD simulations

**Figure 1:**
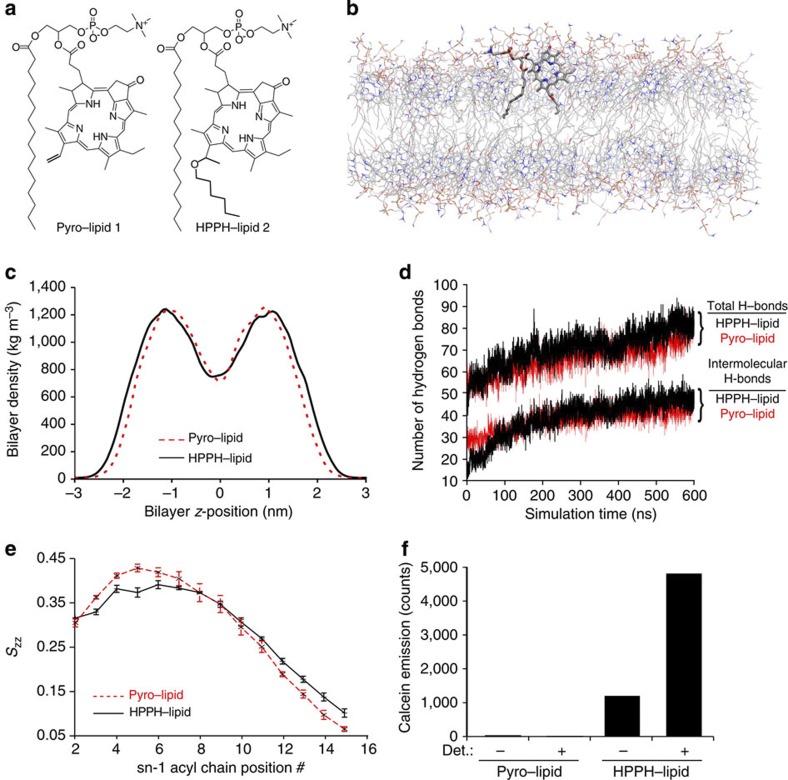
MD simulations of a stable porphyrin bilayer. (**a**) Chemical structures of pyro–lipid 1 and HPPH-lipid 2. Differences are shown in bold. (**b**) Cross-sectional area of bilayers formed entirely from HPPH–lipid in a 500 ns, 128 monomer MD simulation. One of the HPPH monomers is depicted in wire frame format. (**c**) HPPH–lipid and pyro–lipid density (excluding water contribution) post 500 ns MD simulation. (**d**) Evolution of hydrogen bonds formed during MD simulation. Total (intermolecular plus intramolecular) and intermolecular hydrogen bonds for each type of porphyrin–lipid are indicated. (**e**) Chain order parameter (*S*_ZZ_) for porphyrin–lipids following 500 ns MD simulation. *S*_ZZ_ indicates order of lipid chain with respect to bilayer normal vector. Error bars show range for last two adjacent 50 ns block means. (**f**) Stable cargo retention from bilayers of HPPH–lipid, but not pyro–lipid. Both monomers were synthesized and assembled into nanovesicles loaded with calcein at self-quenching concentrations. Nanovesicles were separated from unentrapped calcein before assessing fluorescence. Fluorescence emission of retained calcein in nanovesicles is shown prior or following addition of detergent (det.; 0.25% Triton X-100).

We recently discovered that PoP can self-assemble into liposome-like porphysome nanovesicles formed entirely from a porphyrin bilayer with intrinsic biophotonic character, nanoscale optical properties, biocompatibility and biodegradability^27^,^28^,^29^. However, the previously described porphysome monomers could not assemble into nanovesicles that stably load and retain cargo without addition of cholesterol. To overcome this shortcoming, we examined the structure of the previously developed sn-1-palmitoyl sn-2-pyropheophorbide phosphtatidylcholine (pyro–lipid) monomer 1 (Fig. 1a), and qualitatively noticed an apparent discrepancy between the length of the alkyl side chain and the adjacent porphyrin structure. We hypothesized that devinyl hexyloxyethy-pyropheophorbide (HPPH)-based monomers might form bilayers with superior self-assembly and packing properties because of space filling by the hexyl ether moiety (Fig. 1a). HPPH was selected as it is not only a simple derivative of pyro, but it also has been safely used in numerous human clinical trials^30^. Thus, upon eventual ester hydrolysis of the monomer, both breakdown products of the lysolipid and HPPH would be clinically approved molecules.

To assess the hypothesis that HPPH-lipid 2 (Fig. 1a)-based nanovesicles might enable better cargo loading, we used MD simulations that have been shown to be useful for determining molecular and supramolecular physical properties of lipid bilayers^31^,^32^. We modified the existing porphyrin and lipid force fields^33^ to generate the porphyrin–lipid parameters and performed MD simulations with a bilayer system composed of 128 molecules of either pyro–lipid or HPPH–lipid. Water was added to produce a 3 nm layer between periodic images of the membrane that required an average of 9,640 water molecules per system. Figure 1b shows a cross-section of the HPPH–lipid bilayer with the structures of one of the monomers shown in bold. As shown in Fig. 1c, following a 500 ns simulation, the bilayer density plot revealed that the HPPH–lipid did not give rise to a bilayer with greater maximum density, but rather a thicker bilayer (3.2 versus 2.9 nm with the Gibbs–Luzzati criterion). MD simulations (500 ns) showed that the hexyl ether moiety provides space filling between the two bilayer leaflets compared with the pyro–lipid bilayer that had the central portion of the bilayer filled only with palmitoyl chains (Supplementary Movie 1). Supplementary Figure 1 depicts an example of intramolecular and intermolecular hydrogen bonding in the HPPH–lipid bilayer. Both pyro–lipid and HPPH–lipid contain two hydrogen bond donors located in the porphyrin ring, and hydrogen bond acceptors located both in the porphyrin ring and the oxygens in the esters of the glycerol backbone, and the oxygens on the phosphate. As shown in Fig. 1d, both pyro–lipid and HPPH–lipid formed bilayers with an approximate equivalent amount of total hydrogen bonds. However, out to 600 ns, there was a slight but noticeable trend of increasing intermolecular hydrogen bonds within the HPPH–lipid bilayer, which might generate additional stability within the bilayer. The chain order parameter (*S*_ZZ_) indicates the orientation of the lipid chain with respect to the bilayer normal. Values near 1 indicate an average orientation parallel to the bilayer normal and values closer to 0 (zero) indicate an orientation angled close to 45 degrees away from bilayer normal. The terminal positions of the sn-1-palmitoyl chains in HPPH–lipid bilayers had less disorder than pyro–lipid bilayers as shown in Fig. 1e. Thus, MD simulations suggested that HPPH–lipid bilayers could possibly have enhanced stability compared with pyro–lipid bilayers owing to a slightly thicker bilayer, enhanced intermolecular hydrogen bonding, and more order in the terminal portions of the palmitoyl side chains. We successfully synthesize HPPH–lipid and experimentally examined the behaviour of nanovesicles formed from either pyro–lipid or HPPH–lipid, along with 5 mol. % polyethylene glycol (PEG)-lipid to enhance physiological properties^34^. As previously observed^27^, pyro–lipid nanovesicles hydrated with a 100 mM calcein solution could not stably retain the fluorophore and it was not detectable following nanovesicle isolation (Fig. 1d). However, nanovesicles formed from HPPH–lipid entrapped calcein with high retention efficacy so that it remained self-quenched in the nanovesicles before permeabilization with 0.25% Triton X-100 detergent. Thus, MD simulations correctly predicted a more stable bilayer from HPPH–lipid, which was demonstrated experimentally to enable robust entrapment of cargo inside the nanovesicles.

### Light-triggered permeabilization of PoP-liposomes

**Figure 2:**
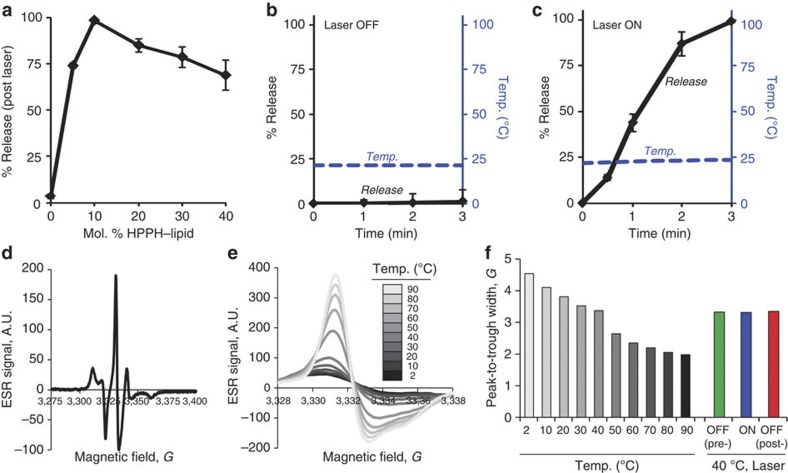
NIR-mediated liposomal cargo unloading in the absence of heating. (**a**) Calcein-loaded PoP-liposomes were formed from 5% PEG–lipid, 35% cholesterol and 60% DSPC. HPPH–lipid was titrated in place of DSPC as indicated. Liposomes were irradiated for 3 min with a 120 mW 658 nm laser and release was assessed. Mean±s.d. for *n*=3. (**b**,**c**) Calcein release (solid black line) and solution temperature (Temp; dashed blue line) was measured for PoP-liposomes in the absence (**b**) or presence (**c**) of 150 mW laser irradiation. Temperature in the solution was measured using a thermocouple. (**d**) ESR of a PoP-liposome sample containing 1 mol. % 5-DSA as a spin label, recorded at 50 °C. (**e**) Temperature dependence of ESR spectra of 5-DSA containing PoP-liposomes. (**f**) Evidence for lack of nanoscale heating in irradiated PoP-liposomes. The central ESR peak-to-trough width is shown for PoP-liposomes containing 5-DSA at various temperature and before (green), during (blue) and after (red) irradiation that induces permeabilization.

With stable cargo loading made possible by the development of HPPH–lipid, we next set out to assess whether NIR irradiation at the specific absorption peak of HPPH could cause the release of loaded cargo. Calcein was entrapped at self-quenching concentrations, and a 658 nm laser was used to irradiate samples for 3 min at 120 mW (240 mW cm^−2^ power density). A starting point of 5% distearoylphosphatidylethanolamine-PEG 2000 (PEG–lipid), 35% cholesterol and 60% 1,2-distearoyl-sn-glycero-3-phosphocholine (DSPC) was selected, as this formulation is similar to stable and clinically proven liposomal doxorubicin (Dox) formulations^35^, and then DSPC was incrementally replaced with HPPH–lipid. Without any HPPH–lipid doping, the liposomes remained fully loaded following laser irradiation. However, when only 10 mol. % HPPH–lipid was included, complete cargo release was observed following irradiation (Fig. 2a). Unexpectedly, as a greater portion of HPPH–lipid was titrated into the PoP-liposomes, the amount of light-induced permeabilization decreased, despite the higher optical character of the bilayer. Thus, beyond an optimal portion, HPPH–lipid had a stabilizing effect on cargo retention in PoP-liposomes in response to laser irradiation. The kinetics of NIR laser-induced release were examined while simultaneously monitoring the solution temperature. In the absence of laser irradiation, the solution temperature remained constant at room temperature and there was no cargo release (Fig. 2b). As PoP-liposomes were exposed to NIR irradiation, cargo was released steadily and completely over the course of 3 min (Fig. 2c). Release from PoP-liposomes occurred without any significant increase in the solution temperature. This was unexpected and represents a departure from conventional triggered liposomal release mechanisms that rely on solution heating to trigger phase transitions. Although no heating was observed based on inserted thermocouple measurements, heating occurring directly at the PoP-liposome bilayer could not be ruled out based on bulk solution measurements alone. To assess local heating, we incorporated 1 mol. % of 5-doxyl steric acid spin label (5-DSA), a commonly used electron spin resonance (ESR) probe that measures temperature-related bilayer fluidity based on probe tumbling rate^36^. 5-DSA that was incorporated into PoP-liposomes produced a characteristic ESR spectrum with one central peak flanked by smaller ones (Fig. 2d). As shown in Fig. 2e, the peak width of the central feature narrowed at elevated temperatures, as is expected for a nitroxide spin label because of increased tumbling rates. Thus, 5-DSA provided a means to assess nanoscale heating during laser irradiation. By measuring the peak-to-trough width of the central spectral feature, nanoscale thermal analysis of bilayer heating during laser irradiation was possible (Fig. 2f). Based on thermally calibrated ESR measurements, during laser irradiation conditions that permeabilized the PoP-liposomes, there was no appreciable bilayer heating (a change of <0.5 °C).

**Figure 3:**
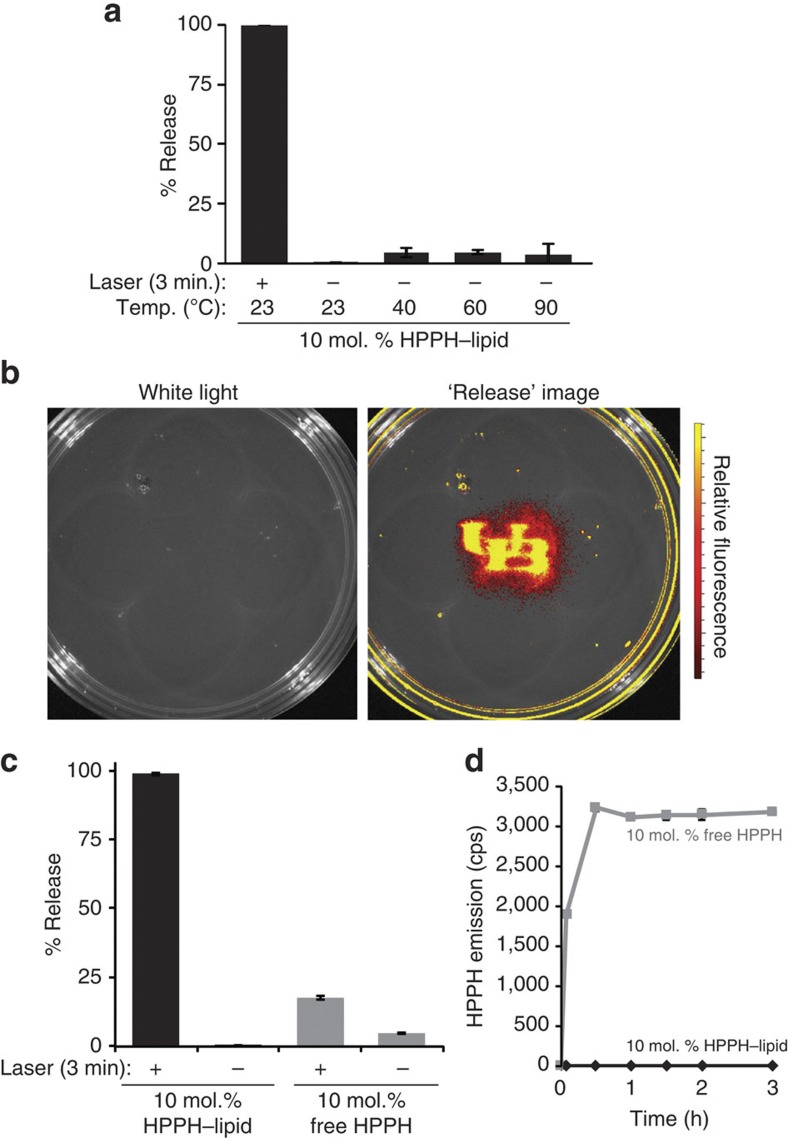
Stability of PoP-liposomes. (**a**) Calcein-loaded PoP-liposomes doped with 10 molar (mol.) % HPPH–lipid were incubated for 10 min in saline at the indicated temperatures with or without a 3 min laser pre-treatment. Mean±s.d. for *n*=3. (**b**) Sulforhodamine B-loaded PoP-liposomes were added to hot agarose (~60 °C) before pouring and solidification. A laser was used to mediate cargo release with high spatial control and spell ‘UB’. Note the dye is distributed equally everywhere in the agarose. (**c**) Calcein release in liposomes formed with 10 mol. percent HPPH–lipid or free HPPH and irradiated with light. Mean±s.d. for *n*=3. (**d**) Rapid serum redistribution of liposomes containing free HPPH, but not HPPH–lipid, as judged by fluorescence unquenching. Mean±s.d. for *n*=3.

As NIR-induced permeabilization was not driven by laser-induced bulk or nanoscale heating effects, the thermal stability of PoP-liposomes in externally heated solutions was examined. Previously, it has been shown that porphyrin–lipid and cholesterol–lipid conjugates attenuate temperature-induced lipid bilayer phase transitions (that destabilize membranes)^27^,^37^. As shown in Fig. 3a, in the absence of irradiation, PoP-liposomes could stably retain their loaded cargo at 40, 60 and even 90 °C. This provides further evidence that a temperature-related phase transition-based mechanism is not responsible for the light-induced release. The thermostability enabled PoP-liposomes loaded with sulforhodamine B to be incorporated into hot agarose (~50 °C) before solidification without leakage. A 658 nm laser could be used to achieve excellent spatial control of permeabilization and release of sulforhodamine B within the solidified agarose (Fig. 3b). To gain insight on the nature of the light-induced release, we examined whether HPPH–lipid was specifically contributing to release via concerted supramolecular effects, or whether HPPH–lipid was acting simply as a light-absorbing photothermal transducing component within the bilayer. We previously found that liposome formation becomes physically impossible and large aggregation occurs when >15 mol. % free porphyrin is included into the formulation^27^. However, because optimal doping in PoP-liposomes used only 10 mol. % porphyrin, it was possible to directly compare liposomes composed identically except for the inclusion of 10 mol. % of either free HPPH or HPPH–lipid. Gel filtration demonstrated that free HPPH, which has minimal water solubility, fully incorporated into the liposomes (Supplementary Fig. 2). Both types of liposomes could be formed and stably entrapped calcein. Both types of liposomes exhibited HPPH fluorescence quenching of >90% compared with the detergent-disrupted liposomes (Supplementary Fig. 3), creating a comparative system as fluorescence self-quenching is correlated to downstream events such as quenching of singlet oxygen generation^38^. When irradiated with NIR light, liposomes containing free HPPH displayed limited light-induced release and PoP-liposomes were over five times more effective at releasing cargo (Fig. 3c). Considering the high background leakage rate of liposomes containing free HPPH, PoP-liposomes were 150 times more effective with respect to the ratio of release following laser irradiation to release in the absence of triggering stimuli. This demonstrates that the constraints conferred by the HPPH–lipid within the porphyrin bilayer have an active role in the release. The presence of 10 mol. % HPPH–lipid in the bilayer did not inhibit the solubilization of a membrane-partitioning drug, amphotericin B, into a clinically used formulation^35^ of that drug (Supplementary Fig. 4). Despite the limited light-triggering efficacy of free HPPH, in theory any NIR chromophore (including free HPPH) could be loaded into liposome bilayers and be used to induce some degree of light-responsive release. However, the physiological significance of such an approach is unclear, because when incubated with serum, hydrophobic molecules embedded in liposomes rapidly equilibrate with lipophilic serum proteins. Indeed, when incubated with serum, free HPPH incorporated into liposomes rapidly transferred to serum components and became completely fluorescently unquenched within 30 min (Fig. 3d). In contrast, PoP-liposomes remained self-quenched, indicating a lack of detectable exchange of HPPH–lipid with serum components. Following intravenous (i.v.) administration in BALB/c mice, PoP-liposomes exhibited a long single compartment circulation half-life of 14.4 h (Supplementary Fig. 5). This is similar to previously observed 12–13 h one compartment circulation half-life of porphysome nanovesicles containing 60 mol. % pyro–lipid^28^, yet somewhat shorter than some formulations of sterically stabilized PEGylated liposomes for Dox delivery, which can exhibit 20 h circulation half-lives in mice^39^.

### Spatial and temporal control of cargo release

**Figure 4:**
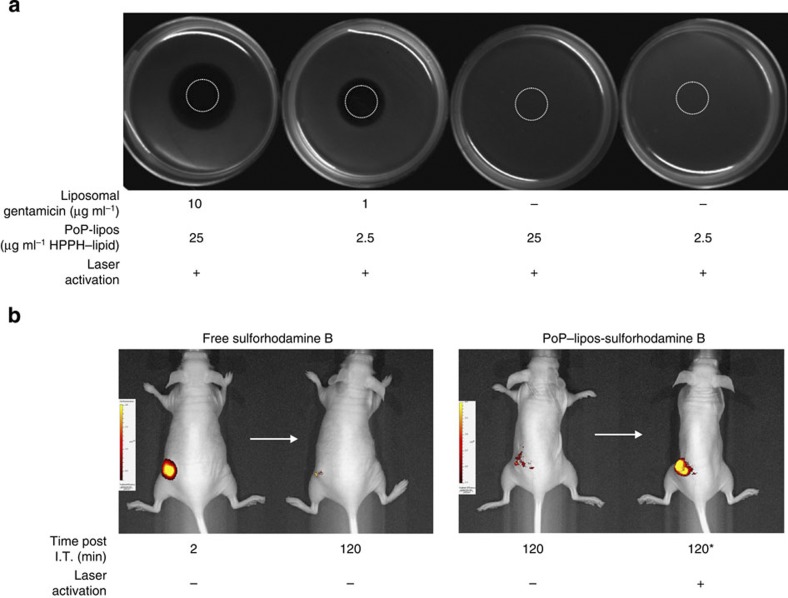
Spatial and temporal control of PoP-liposome permeabilization. (**a**) Spatial control of *B. subtilis* killing with triggered antibiotic release. Gentamicin was loaded in PoP-liposomes and embedded in hot agar along with the bacteria. The indicated spot was irradiated with a 658 nm laser (200 mW cm^−2^) for 10 min and the plates were photographed 24 h later. (**b**) Temporal control of cargo release in Panc-1 xenografts. Mice were imaged following intratumoral (I.T.) injection of 5 nmol of either free sulforhodamine B or sulforhodamine B entrapped at self-quenching concentrations in PoP-liposomes. Representative images are shown of the indicated time points and conditions. Laser activation was performed after 2 h. Representative results shown with *n*=3 mice per treatment group.

To emphasize spatial and temporal control of release and demonstrate wide-ranging utility of PoP-liposomes, we developed distinct proof-of-principal experiments for antibacterial and antineoplastic applications. Liposomes have attractive properties for antibacterial drug delivery^40^, and gentamicin, a common aminoglycoside antibiotic, could be passively loaded into the interior of PoP-liposomes. Owing to their thermal stability, gentamicin–PoP-liposomes could be impregnated into hot agar before solidification along with *Bacillus subtilis*, a model Gram-positive bacteria. As shown in Fig. 4a, when impregnated with 10 μg ml^−1^ gentamicin–PoP-liposomes (based on gentamicin concentration), specific drug release in the agar occurred upon laser exposure and complete bacteria killing was achieved within the irradiated spot. In addition, a zone of inhibition 6 mm outside the irradiated area was also observed because of drug diffusion within the agar. Significantly, the non-irradiated areas outside the zone of inhibition demonstrated heavy bacterial growth, despite the presence of equally high concentrations of (entrapped) gentamicin throughout the agar. Because even a 10-fold dilution of the gentamicin–PoP-liposomes maintained light-triggered killing (Fig. 4a), the lack of cell killing in the non-irradiated areas of the high concentration dish reflects the stable entrapment efficacy of PoP-liposomes. Control agar plates impregnated with empty PoP-liposomes did not show significant light-triggered antibacterial effect. Thus, gentamicin–PoP-liposomes demonstrated efficient spatial control of antibiotic release that may not be achievable as easily or directly using any other triggered release methods. To demonstrate a unique application for temporal control of cargo release, we injected either free sulforhodamine B or sulforhodamine B–PoP-liposomes intratumorally into nude mice bearing subcutaneous Panc-1 xenografts. Owing to their larger size relative to small molecules, liposomes do not rapidly drain from tumours following direct injection, and have demonstrated potential as intratumoral drug delivery vehicles^41^. PoP-liposomes could add a new layer of control to trigger liposomal release following liposomal redistribution within the tumours. As seen in Fig. 4b, following intratumoral injection of free rhodamine, the molecule rapidly drained from the tumour, with little fluorescence remaining in the tumour 2 h following injection. This was not surprising as small molecule fluorophores are known drain to lymph nodes within minutes^42^, and previous studies of intratumoral injection of methylene blue revealed rapid drainage kinetics^43^. Larger nanoparticles (100–200 nm) can be used to significantly slow down the rate of drainage on the order of hours rather than minutes^44^. Sulforhodamine B–PoP-liposomes fall in this size range and were formed with self-quenching concentrations of the fluorophore. Two hours following intratumoral injection, the tumour was irradiated, and released sulforhodamine B could be clearly visualized following its unquenching. This demonstrated that not only did the size of the liposomes modulate tumour distribution following intratumoral injection, but that the PoP-liposomes were sufficiently stable *in vivo* to permit release of contents only after light treatment following a 2 h incubation period.

### Dox loading and release

**Figure 5:**
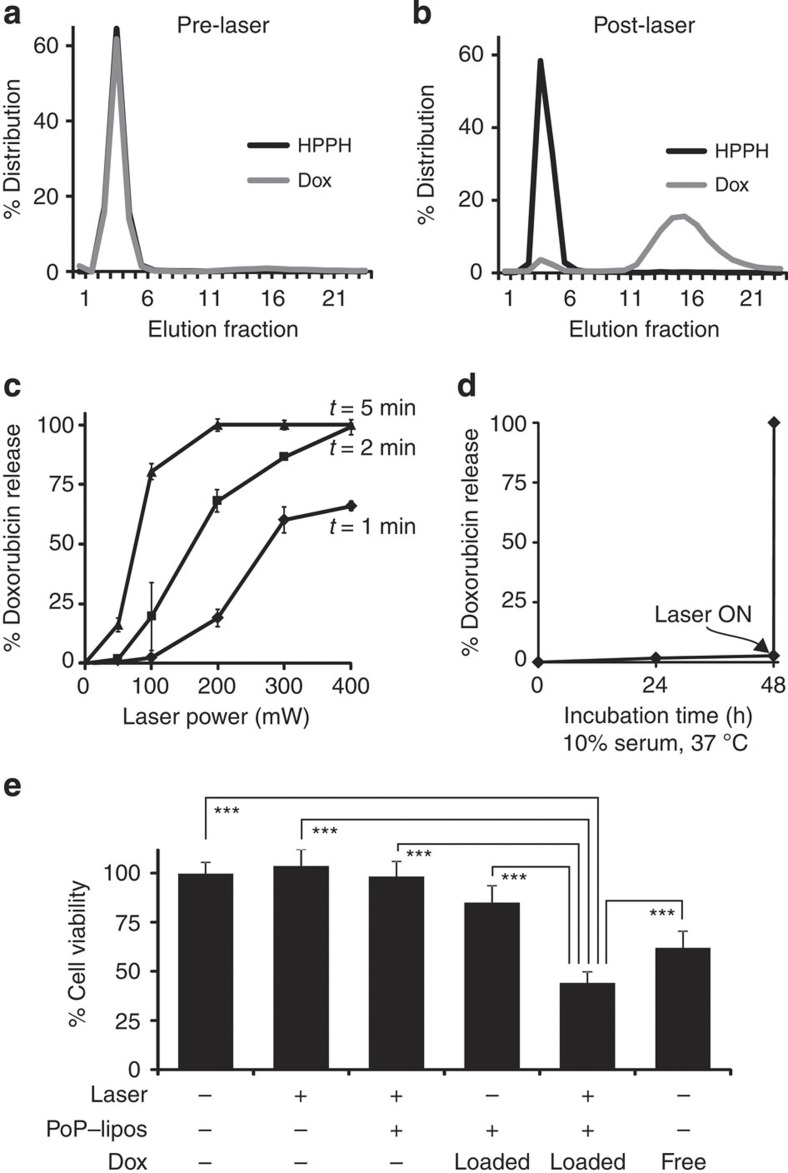
Tunable and on-demand release of Dox in PoP-liposomes. (**a**) Gel filtration demonstrating active Dox loading in PoP-liposomes. Over 95% of the Dox was loaded in Dox–PoP-liposomes using a 10:1 lipid to drug ratio at 60 °C for 1 h. (**b**) Gel filtration of liposomes following laser irradiation showing effective light-triggered release. (**c**) Tunable drug release using PoP-liposomes. Dox–PoP-liposomes were irradiated at varying times and laser powers in media containing 10% serum. Release was assessed using fluorescence. Mean±s.d. for *n*=3. (**d**) Stability of Dox–PoP-liposomes incubated in 10% serum at 37 °C for 2 days and subsequently subjected to 4 min, 300 mW laser irradiation. Mean±s.d. for *n*=3. (**e**) *In vitro* cell killing using Dox–PoP-liposomes. Panc-1 cells were incubated as indicated for 24 h with 10 μg ml^−1^ Dox following exposure to 200 mW cm^−2^ irradiation for 10 min. After 24 h, the media was replaced and viability was assessed 24 h later using the XTT assay. Mean±s.d. for *n*=8. ***Laser+Dox–PoP-liposomes induced significant inhibition of cell viability compared with all other groups based on one-way analysis of variance with *post hoc* Tukey’s test (*P*<0.001).

Active liposomal drug loading makes use of ion and pH gradients to concentrate drugs into the aqueous interior of liposomes. We examined whether active Dox loading was possible using PoP-liposomes, given the stabilizing nature of the porphyrin-doped bilayer. As shown from the gel filtration results in Fig. 5a, Dox could be actively loaded with >95% efficacy following incubation at 60 °C for 1 h using an internal ammonium sulphate gradient. Figure 5b shows that following irradiation, Dox, but not HPPH–lipid, was released from the PoP-liposomes. When Dox–PoP-liposomes were irradiated in a saline buffer including 10% fetal bovine serum, release occurred in both a laser power and irradiation time dose-dependent manner (Fig. 5c). It is noteworthy that in addition to varying release characteristics by modulating porphyrin doping and irradiation time, laser power could directly affect release. Over a wide range of fluence rates, Dox release increased with increasing irradiation times (Supplementary Fig. 6a). Unlike photodynamic therapy, in which higher fluence rates yield lower effects because of depletion of molecular oxygen, complete Dox release from PoP-liposomes depended on total fluence (~85 J cm^−2^), and the time required for full release could be predicted as a function of fluence rate (Supplementary Fig. 6b). Unlike phase transition-based mechanisms that are constrained to heating target areas to set temperatures, laser power variation adds a powerful new layer of direct control to triggered release. This might be exploited by controlling release into microvessels of specific target sizes, as when PoP-liposomes were irradiated in flowing capillary tubing, release was dependent on solution velocity (Supplementary Fig. 7). Controlled release has upside for Dox in particular, as clinical liposomal Dox formulations have been shown to have a slow release rate that results in only 50% bioavailability even after 1 week *in vivo*, thereby limiting therapeutic efficacy^45^. Figure 5d demonstrates that *in vitro*, there was minimal release from Dox-loaded PoP-liposomes throughout the course of a 48-h incubation in physiological conditions. However, when the sample was subjected to 300 mW, 658 nm laser irradiation, complete release occurred in just 4 min. This compares favourably to phase transition-based systems such as thermally triggered liposomes, which generally exhibit a ~10-fold acceleration in release in physiological conditions when comparing the ‘on’ versus ‘off’ states^11^. Figure 5e demonstrates that Dox–PoP-liposomes could be used to induce light-triggered inhibition of viability of Panc-1 cells. Laser treatment in the presence or absence of empty PoP-liposomes had no effect on cell viability. Without light treatment, Dox–PoP-liposomes, incubated with the cells for 24 h in 10% serum at 37 °C, induced a small amount (~15%) of inhibition of cell viability. This was anticipated because of some uptake of the drug-loaded liposomes in the extended duration incubation. However, when the Dox–PoP-liposomes were treated with 200 mW cm^−2^ for 5 min, significant inhibition (>50%) of cell viability was observed, which was even more effective than the free drug. The reason for superior efficacy of the released Dox compared with the free drug is not clear, but one explanation may be that the free drug bound to serum proteins that impacted cellular uptake, whereas the presence of PoP-liposomes may have interfered with that process. Confocal microscopy revealed that light-triggered release of Dox–PoP-liposomes resulted in the Dox becoming bioavailable and translocating to the cell nucleus of Panc-1 pancreatic cancer cells when incubated in serum for 3 h (Supplementary Fig. 8).

**Figure 6:**
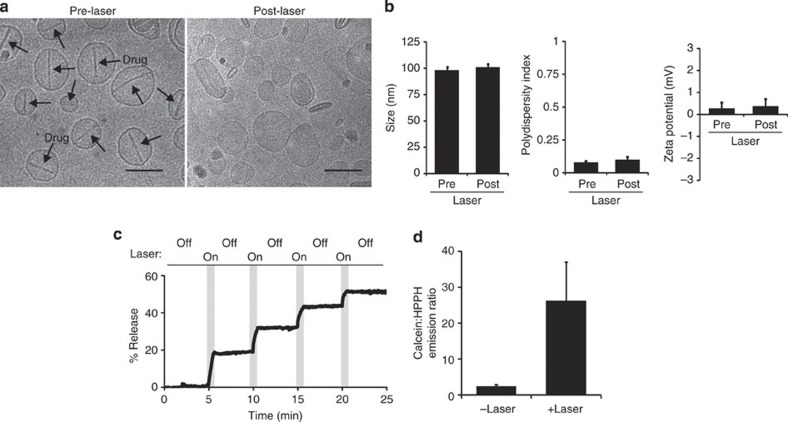
Insights into light-induced transient permeabilization of PoP-liposomes. (**a**) Representative cryo-TEM images of Dox–PoP-liposomes before and after irradiation. Arrows indicate the formation of Dox-sulphate crystals within the liposomes. Scale bars, 100 nm. (**b**) Dynamic light scattering size, polydispersity index and zeta potential before and after laser-induced release of Dox–PoP-liposomes. (**c**) Temporary induced permeability as demonstrated by periodic laser irradiation of calcein-loaded PoP-liposomes. (**d**) Empty PoP-liposomes were incubated in a 2 mM calcein solution and irradiated with 120 mW laser irradiation for 3 min. Free calcein and PoP-liposome-entrapped calcein were separated with gel filtration, and the ratio of calcein emission to HPPH emission (using separate excitation and emission settings) was measured. Mean±s.d. for *n*=3.

We next elucidated mechanistic insights into the properties of light-triggered release. Cryogenic transmission electron microscopy (cryo-TEM) revealed that Dox–PoP-liposomes contained characteristic fibrous sulphate-Dox crystals within their core (Fig. 6a). Following laser irradiation, not only was Dox released from the PoP-liposomes, but they clearly re-formed with an intact bilayer, providing direct evidence for the propensity for the PoP-liposomes to open and then close in response to NIR light. Further confirming a non-destructive permeabilization mechanism, no change in liposome size, polydispersity and zeta potential was observed following NIR irradiation of Dox–PoP-liposomes (Fig. 6b). To test the hypothesis that membranes stably resealed following irradiation, calcein-loaded PoP-liposomes were irradiated intermittently, and release during laser ‘on’ and ‘off’ periods was examined in real time. As shown in Fig. 6c, calcein release from liposomes occurred only during irradiation and ceased within seconds of turning the laser off. Negligible release was observed in the periods without laser irradiation. This suggests that PoP-liposomes rapidly resealed and re-formed stable bilayers as soon as irradiation was halted, as if permanent destabilization was occurring some level of background release would be expected immediately following laser stoppage. This provides evidence against a release mechanism that is based on covalent chemical photoreaction within the bilayer. Further evidence is shown in Fig. 6e, which demonstrates that NIR laser exposure could be used to induce temporary permeability, with liposomes able to reseal and entrap external contents. This would not be possible if the laser was inducing reactions that caused permanent lipid bilayer instability. When empty PoP-liposomes were placed in a calcein-containing solution and subjected to laser irradiation, calcein could diffuse into the liposomes and be stably retained there, demonstrating that transient permeabilization could be used to allow loading (as opposed to release) of cargo. Only minimal loading was achieved (0.012% of external calcein), although this could be increased by increasing PoP-liposomes concentration. Under these conditions, minimal (~5%) photobleaching was observed. This represents a novel method for loading of cargo on demand.

### Antitumour therapy

**Figure 7:**
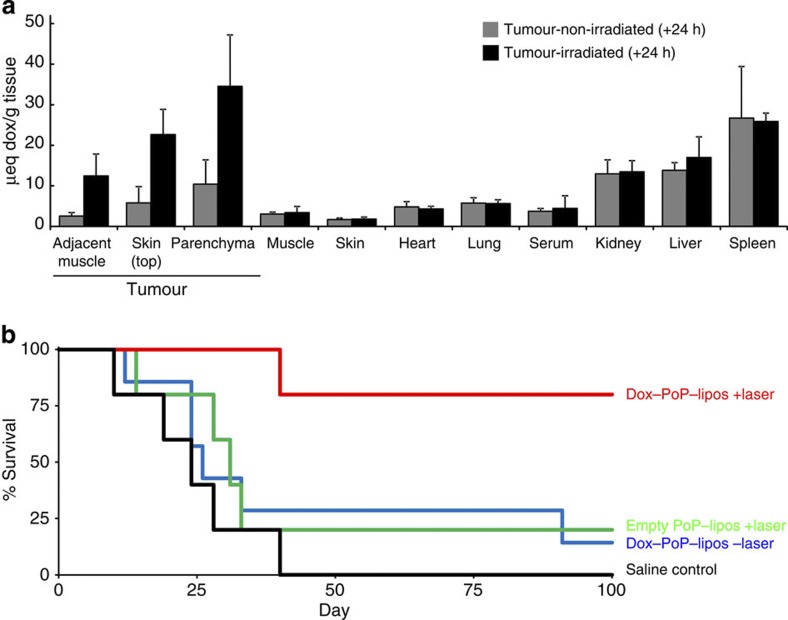
Dox–PoP-liposome antitumour phototherapy. (**a**) Biodistribution of Dox± laser treatment. Nude mice bearing KB tumours were i.v. injected with Dox–PoP-liposomes (10 mg kg^−1^ Dox) and 15 min later, the tumour was irradiated with a 658 nm laser at 200 mW cm^−2^ fluence rate for 12.5 min (150 J cm^−2^). Mean±s.d. for *n*=7–8 mice per group. Comparing laser-treated mice to non-laser-treated mice with a two-tailed Student’s independent sample *t*-test, only the tumour-associated tissues had statistically significant differences in Dox accumulation (*P*<0.05). (**b**) Kaplan–Meier survival curve for nude mice bearing KB tumours. Mice were given a single treatment when tumours reached 4–6 mm and were killed when tumours reached 10 mm in any direction. Mice were treated with Dox–PoP-liposomes (10 mg kg^−1^ Dox) as above or a corresponding amount of empty PoP-liposomes. *n*=5–7 mice per group. Based on the log-rank test, there was a statistically significant difference between the various treatment groups; and pairwise comparisons showed that Dox–PoP-lipos+laser group lived significantly longer than each other group (*P*<0.05).

The potential utility of Dox–PoP-liposomes for systemically administered therapy was assessed in nude mice bearing subcutaneous tumors grown from the KB cancer cell line. Mice were injected with doses of 10 mg kg^−1^ Dox–PoP-liposomes. Fifteen minutes following i.v. injection, tumours were irradiated with 658 nm laser light at 200 mW cm^−2^ for 12.5 min (150 J cm^−2^), or were not irradiated as a control. Twenty-four hours later, organs were collected and Dox biodistribution was assessed. As shown in Fig. 7a, laser treatment resulted in the deposition of about threefold more Dox compared with non-irradiated tumours, whereas none of the other organs displayed statistically significant differences. Significant enhancement of Dox deposition was noted in other laser-irradiated tissues such as in the skin covering the tumour as well as in muscle that was immediately adjacent to the tumour. This underscores clinical challenges that will necessitate careful light delivery specifically to the tumour and not to adjacent critical organs. Although intratumoral injection followed by irradiation (Fig. 4b) definitively demonstrated that PoP-liposomes are capable of light-triggered release *in vivo*, the biodistribution results are more complex to interpret. Besides direct drug release, the observed enhancement in tumour Dox deposition could have been influenced by other factors including enhanced tumour permeability due to vascular damage induced by initial drug release and also from photosensitizing effects of the PoP-liposomes during irradiation. It has been demonstrated that photodynamic therapy (PDT) using HPPH led to enhanced tumour deposition of liposomal Dox in a murine colon carcinoma model^46^. Future studies will seek to better understand how these multiple factors contribute to enhanced tumour deposition of Dox in PoP-liposomes. Based on the favourable biodistribution results, we performed a survival study based on a single i.v. treatment of Dox–PoP-liposomes (10 mg kg^−1^ Dox dose). Mice were divided into four groups as follows: (1) Dox–PoP-liposomes with laser treatment; (2) empty PoP-liposomes with laser treatment; (3) Dox–PoP-liposomes without laser treatment; and (4) saline control. As shown in Fig. 7b, only the Dox–PoP-liposomes with laser treatment effectively cured tumours. Four out of the five mice treated had tumours permanently cured with no regrowth after 90 days. Following laser treatment, both Dox–PoP-liposomes and empty PoP-liposomes induced eschar formation on the skin of the tumour, demonstrating that PoP-liposomes themselves have photosensitizing properties. However, the irradiated empty PoP-liposomes were inneffective at halting tumour growth. Likewise, a single dose of Dox–PoP-liposomes without laser treatment was also ineffective at halting tumour growth. Thus, only the laser treatment of Dox–PoP-liposomes was an effective treatment resulting in complete tumour regressions. Although no treated mice exhibited signs of phototoxicty under ambient lighting (no special housing precautions were taken), and PoP-liposomes are self-quenched while intact, their eventual disassembly and degradation following systemic introduction could lead to restoration in HPPH photosensitizer activity, which is known to induce mild skin photosensitivity in clinical use^47^.

## Discussion

PoP-liposomes are distinguished from our previously described porphysome nanovesicles by containing a relatively small amount of PoP, only 10 mol. %. As such, they more closely resemble a modified liposome rather than a porphysome. The nature of the NIR-induced permeabilization is not well understood and further work must seek to better characterize both its chemical and physical mechanism. Optical microscopy has recently been shown to be useful for observing light-induced permeabilization in porphyrin–lipid microvesicles^48^, although mechanistic aspects between nanoscale and microscale vesicle permeabilization are likely different. Although the *in vivo* results of the systemically administered Dox–PoP-liposomes clearly demonstrated the synergistic effects of light treatment, further investigation is required to characterize the mechanism of action in the tumour.

In summary, PoP-liposomes assembled from appropriate porphyrin–lipid monomers formed a robust system that achieved thermostable cargo retention as well as effective release upon exposure to clinically relevant doses of NIR radiation. Release could be tuned by varying porphyrin doping, laser irradiation time and laser irradiation power. This represents a departure from most other proposed biocompatible externally triggered release systems that rely on heating to a few degrees above body temperature. In response to NIR irradiation, PoP-liposomes released their cargo with robust spatial and temporal control and when loaded with Dox could be used in an effective antitumour phototherapy.

## Methods

### MD simulations

Membranes made of two different porphyrin–lipid molecules were simulated using MD. Each bilayer system was composed of 128 molecules of either the pyro or HPPH variant of the porphyrin–lipids. Water molecules (9,640) were added to achieve full hydration. The GROMACS software package was used^49^. Starting with the standard united atom force field, the lipid models were defined by removing the sn-2 tail from dipalmitoyl phosphatidylcholine and replacing it with the haem available in the force-field database. GROMOS 53a6 force-field^33^ was used to build the final structures and to modify the haem. This force-field has been shown to perform well in simulations of lipids and peptides^50^,^51^. Partial charges were chosen from analogous moities in the force-field database. The simple point charge model was used for water. Bond lengths were constrained using the SETTLE^52^ algorithm for water and LINCS^53^ for the lipids. Lennard–Jones interactions were treated with a switching algorithm that started at 0.8 nm and used a cutoff of 0.9 nm. Electrostatics were treated with the particle-mesh Ewald method^54^ using a real space cutoff of 1.4 nm, beta spline interpolation (of order 4), and a direct sum tolerance of 1E-6. Periodic boundaries were used in all three dimensions and a time step of 2 fs was used. The systems were set up by arranging 64 lipids per leaflet on a rectangular lattice. Steepest decent energy minimization was done before adding water and then repeated. This was followed by 100 ps of NVT (constant particle number, volume and temperature) relaxation at 273.15 K using a stochastic dynamics integrator and 100 ps of NpT (constant particle number, pressure and temperature) relaxation at 310.15 K. As the final part of relaxation, a 500 ns NpT simulation was run at 310.15 K and 1 bar. Leaf-frog integrator was used. The production simulations were conducted in the NpT ensemble. Pressure and temperature were fixed at 1 bar and 310.15 K, respectively, using the Parrinello–Rahman algorithm^55^ with a relaxation time of 0.5 ps for pressure and the velocity rescaling algorithm^56^ for temperature coupling with a relaxation time of 0.1 ps. The temperatures of the lipids and water were coupled independently. This simulation protocol has been shown to be reliable for membrane systems^50^.

### Synthesis of PoP-liposomes

Pyro–lipid was synthesized as previously described^27^. HPPH–lipid was synthesized in an analogous manner by esterifying HPPH (purified as previously described^57^) at room temperature with 1-palmitoyl-2-hydroxy-*sn*-glycero-3-phosphocholine (lyso-C16-PC, Avanti, no. 855675 P) using 1-ethyl-3-(3-dimethylaminopropyl)carbodiimide (EDC) and 4-dimethylaminopyridine (Fisher, no. AC14827-0250) in chloroform at a 1:1:2:2 lyso-C16-PC:HPPH:EDC:4-dimethylaminopyridine molar ratio. The resulting HPPH–lipid was purified with diol silica gel and freeze dried in a 80% t-butanol (Sigma, no. 360538), 20% water solution. Purity was confirmed with thin layer chromatography (>95% pure) and identity confirmed with mass spectrometry (expected: 1114.7; found: 1114.7; Supplementary Fig. 9) and 1H nuclear magnetic resonance on a Varian Inova 500 MHz spectrometer (Supplementary Note 1). To generate PoP-liposomes, films were prepared by drying chloroform solutions containing DSPC (Avanti, no. 850365P), 1,2-distearoyl-sn-glycero-3-phosphoethanolamine-N-(methoxy(PEG)-2000 (DSPE-PEG2K); Avanti, no. 880120 P), HPPH–lipid and cholesterol (Avanti, no. 700000P) in the indicated molar ratios. The general formulation for PoP-liposomes was based on 50 mol. % DSPC, 35 mol. % cholesterol, 10 mol. % HPPH–lipid and 5 mol. % DSPE-PEG2K. Liposomes incorporating 10 mol. % free HPPH were formed analogously. The chloroform was evaporated using either a stream of argon gas or a rotary evaporator followed by further drying in a desiccator vacuum chamber. The film was re-hydrated with phosphate-buffered saline (pH 7.4) and sonicated for 30 min.

### Cargo release and loading from PoP-liposomes

Self-quenching dye loading was achieved by hydrating and sonicating PoP-liposomes with a 100 mM calcein solution (Sigma, no. 21030) or sulforhodamine B solution (VWR, no. 89139-502) and subsequent gel filtration separation over a Sephadex G-75 column (VWR, no. 95016-784). Dox loading was achieved by extruding a lipid film with a high-pressure lipid extruder (Northern Lipids) with a 250 mM ammonium sulphate solution. Polycarbonate membranes of 0.2, 0.1 and 0.08 μm pore size were sequentially stacked and solution was passed through the extruder 10 times at 60 °C. Free ammonium sulphate was removed by overnight dialysis in a 10% sucrose solution with 10 mM HEPES, pH 7.4. Dox (LC Labs, no. D-4000) was then loaded by adding a 1:10 ratio of drug:lipid and incubating at 60 °C for 1 h. Free Dox was removed by dialysis. For release experiments, PoP-liposome solutions of 0.5–2 mg ml^−1^ were generally diluted by a factor of 50–100 for calcein experiments, and 10–20 times for Dox release, and light could pass freely through the solution without inner filter effects. Release experiments were performed using a hand-held laser diode outputting 120 mW at 658 nm or a tunable 658 nm 500 mW laser diode (LaserGlow) and irradiations were performed as indicated. When incubated in serum, fetal bovine serum was used (VWR, no. 16777-532). Temperature was measured by inserting a K-type thermocouple probe directly into the solution during irradiation. Cargo release was assessed by measuring the release before and after treatment, including solubilization with 0.25% Triton X-100. Release was calculated using the formula release=(F_FINAL_−F_INIT_)/(F_TX100_−F_INIT_) × 100%. OriginPro 8.5 was used for data fitting where equations are indicated. A Zetasizer ZS90 (Malvern Instruments) was used for dynamic light scattering analysis. For spatial control of release experiments, a 1% agarose gel was doped with sulforhodamine B-loaded PoP-liposomes when the agarose had been melted and cooled to ~60 °C, before solidification. Release was then performed using 658 nm laser irradiation with a preformed mask printed on transparency paper. The agarose was imaged using an IVIS Lumina II system. Cargo release using varying flow speeds was performed in tygon tubing with a 0.3 mm inner diameter and a variable speed syringe pump operating at the indicated speeds (New Era, no. NE-1000). For Amphotericin B loading, Amphotericin B (VWR, no. 97061-610) liposomes were formed using the thin film method using a lipid formulations of the indicated molar ratios composed of 1,2-distearoyl-sn-glycero-3-phospho-(1′-rac-glycerol) (Avanti, no. 840465X), hydrogenated soy phosphatidylcholine (Avanti, no. 840058 P), HPPH–lipid and cholesterol. Following liposome formulation via sonication, solutions were filtered, and the amount of Amphotericin B in the filtrate and pre-filtered solution were determined using fluorescence with 365 nm excitation and 470 nm emission.

### Cell viability experiments

*In vitro* cell studies were performed by seeding 10,000 Panc-1 cells per well in a 96-well plate. Drug (10 μg ml^−1^ Dox in free or PoP-liposomal form and/or an equivalent amount of empty PoP-liposomes based on HPPH–lipid concentration) was added as indicated in media (DMEM, VWR, no. 16777-200) containing 10% fetal bovine serum. The wells were irradiated as indicated using a 658 nm laser with 200 mW cm^−2^ fluence rate for 5 min. Drugs and media were left to incubate in media containing serum without removal for 24 h. After 24 h, media was replaced and cell viability was assessed 24 h later using the XTT assay (VWR, no. 89138-264). Viability was calculated by measuring absorption at 450 nm subtracting a 630 nm background reading and normalizing to untreated control cells using a Safire plate reader (TECAN).

### Cryo-electron microscopy

To perform the cryo-EM experiments, 3.4 μl of sample were deposited in a c-flat grids (CF-2/2-2C) with an additional continuous layer of thin carbon (5–10 nm). Grids were glow discharged in air at 5 mA for 15 s before sample addition. Samples contained the Dox–PoP-liposomes before and after irradiation at a concentration of 5 mg ml^−1^ (Dox concentration ~1.5 mg ml^−1^) in a buffer with 10 mM HEPES (pH 7.4) and 10% sucrose. Grids were blotted twice and vitrified by rapidly plunging them into liquid ethane at −180 °C using a Vitrobot (FEI). The blotting chamber of the Vitrobot was set up at 25 °C and 100% relative humidity. Grids were transferred to a JEOL 2010 F electron microscope operated at 200 kV using a Gatan 914 cryo-holder. Images were recorded on Kodak SO-163 films under low dose conditions (~15–20 e per Å^2^) at a nominal magnification of × 50,000 and a defocus of −5 μm. Electron micrographs were digitized with a step size of 12.7 μm in a Nikon Super Coolscan 9000 scanner producing images with a sampling value of 2.54 Å per pixel.

### Electron spin resonance

PoP-liposomes were formed with the inclusion 1% 5-DSA (Sigma, no. 253634) into the standard formulation using the thin film hydration method. A Bruker ER-200 ESR X-band spectrometer was used with a TE102 rectangular cavity and with a Bruker B-VT-1000 nitrogen flow temperature controller. The ESR frequency was 9.44 GHz, field modulation of 1.9 Gauss and microwave power of 0.64 mW. The sample size was ~20 μl in a 1 mm inner diameter quartz tube at the indicated temperatures. Samples were irradiated using a 658 nm 200 mW laser that was focused on the ESR sample through light slots on the Bruker X-band ESR cavity, which are conditions that induce PoP-liposome permeabilization.

### Confocal microscopy

10,000 Panc-1 cells were seeded in eight-well confocal chamber slides (VWR, no. 43300-774) in DMEM media with 10% serum. Twenty-four hours later, cells were incubated with 10 μg ml^−1^ Dox in either free or PoP-liposomal form in DMEM in 10% serum. Laser treatment was performed as indicated and all wells were incubated for an additional 3 h. Media was replaced and cell imaging was performed using a Zeiss LSM 710 confocal microscope with × 20 objective using 490 nm excitation and 612 nm emission.

### Bacteria-killing experiments

PoP-liposomes formed using the thin film method were passively loaded in a solution of 85 mg ml^−1^ gentamicin sulphate (Fisher, no. BP918-1). Following sonication, non-entrapped gentamicin was removed with gel filtration over a Sephadex G-75 column. Gentamicin concentration in the liposomes was determined using a fluorescent assay as previously reported^58^. *B. subtilis* (Cm+) was grown overnight in liquid LB medium at 37 °C overnight. PoP-liposomes and the liquid bacteria culture were combined with melted LB agar in a volume ratio of 1:30. The temperature of the agar was ~45 °C when with *B. subtilis* and the PoP-liposomes. The plates were poured with ~5 ml of bacteria–drug–agar per plate. Following solidification, the plates were irradiated with a 1.2-cm spot diameter with 200 mW cm^−2^ for 10 min using a 658 nm laser. The plate was photographed 24 h later.

### Liposomal release from tumours following intratumoral administration

Animal experiments were carried out with approval from the University at Buffalo Institutional Animal Care and Use Committee. Panc-1 tumours were grown by injecting 6-week-old female nude mice (Charles River) with 3 × 10^6^ Panc-1 cells in a 1:1 Matrigel dilution (BD Biosciences) in the hind flank of the mice. Following several weeks of growth, the tumours were carefully injected intratumorally with sulforhodamine B–PoP-liposomes. Images were acquired with an *in vivo* fluorescence imager (IVIS Lumina II) at the indicated time points with the mice under isoflurane-induced anaesthesia. The tumour was irradiated for 30 min with a 0.6-cm diameter spot size at 200 mW cm^−2^ power density and mice were imaged again.

### Systemically administered treatments

KB cells (Hela subline) were injected in the right flank of 6-week-old female nude mice (Jackson Labs). When tumour volumes reached 4–6 mm in diameter, Dox–PoP-liposomes (10 mg kg^−1^ Dox) or an equivalent dose of empty PoP-liposomes were injected via tail-vein. Ten to fifteen minutes later, tumours were irradiated for 12.5 min with a 200 mW cm^−2^ laser (150 J cm^−2^). For biodistribution studies, mice were killed 24 h later, tissues were homogenized, extracted overnight in acidic isopropanol and Dox biodistribution was determined via fluorescence measurements with 480 nm excitation and 590 nm emission. For survival studies, tumour size was monitored 2–3 times per week and mice were killed when the tumour grew to 1 cm in any dimension.

For pharmacokinetic analysis, male BALB/c mice were injected via tail-vein with empty PoP-liposomes (15 mg kg^−1^ HPPH–lipid). Small blood volumes were sampled at submandibular and retro-orbital locations at the indicated time points and serum was analysed for HPPH content using fluorescence with 400 nm excitation and 660 nm emission following dilution into a 0.25% Triton X-100 solution to prevent any self-quenching.

## Author contributions

K.A.C., S.S. and J.F.L. conceived the project; M.I.H. and M.K. performed molecular simulation experiments and interpreted the data; K.A.C., S.S. and R.K.P. synthesized and characterized materials; V.M.G. and C.P.S. performed electron paramagnetic resonance experiments and interpreted the data; K.A.C. and W.S. performed cell imaging experiments; K.A.C., G.Z. and B.A.P. performed antibacterial experiments; B.A. and J.O. performed cryo-electron microscopy; K.A.C., D.L., H.H. and J.G. performed *in vivo* experiments and interpreted the data; K.A.C., S.S., M.I.H., M.K. and J.F.L. wrote the manuscript.

## Additional information

**How to cite this article:** Carter, K. A. *et al*. Porphyrin–phospholipid liposomes permeabilized by near-infrared light. *Nat. Commun.* 5:3546 doi: 10.1038/ncomms4546 (2014).

## Supplementary Material

Supplementary Figures and Supplementary Notes Supplementary Figures 1-9 and Supplementary Note 1

Supplementary Movie 1 500 ns simulation of pyro-lipid and HPPH-lipid bilayers.
