# Supplementary material for: Porphyrin–phospholipid liposomes permeabilized by near-infrared light
Source: Nat Commun. 2014 Apr 3;5:3546. doi: 10.1038/ncomms4546 (PMC3988818; doi:10.1038/ncomms4546)
Supplement: Supplementary Figures and Supplementary Notes — Supplementary Figures 1-9 and Supplementary Note 1 [file ncomms4546-s1.pdf]

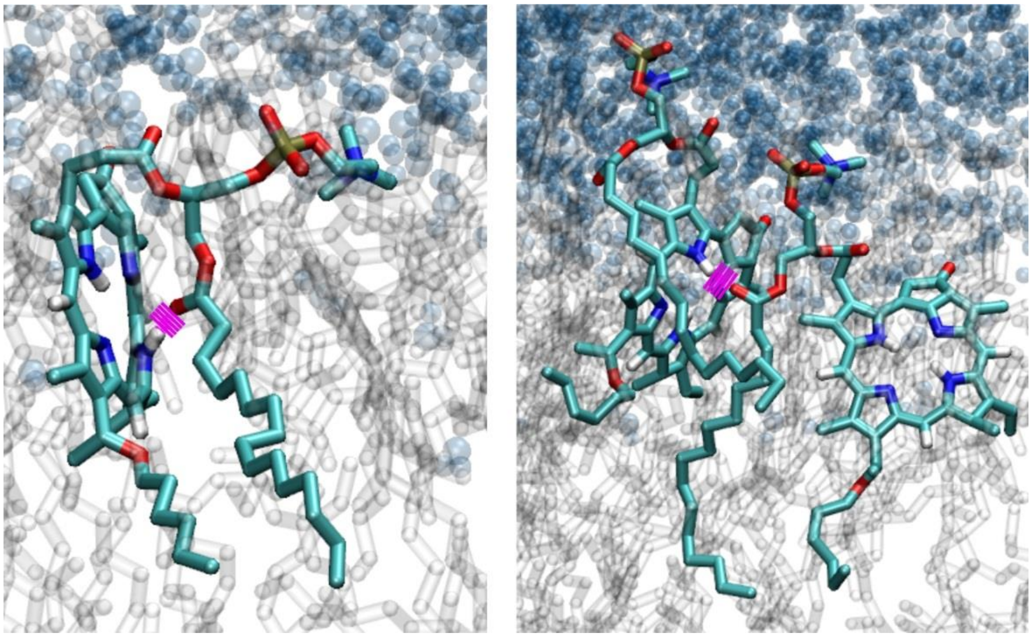

Supplementary Figure 1: Inter- and intra- molecular hydrogen bonding in HPPH-lipid bilayer molecular dynamics simulations. An intramolecular hydrogen bond is shown in magenta between the porphyrin ring of HPPH-lipid and the glycerol backbone (left panel). An intermolecular bond is shown in magenta between two HPPH-phospholipid monomers (right panel).

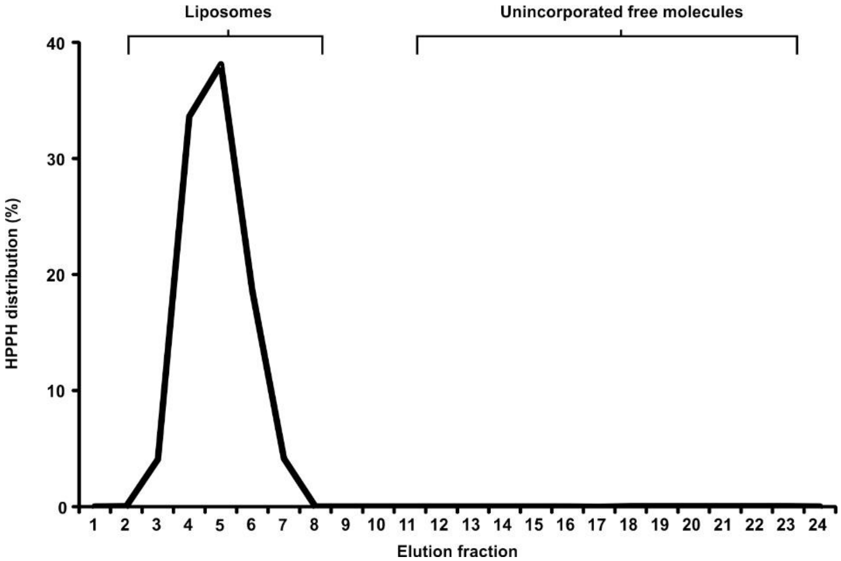

Supplementary Figure 2: Effective loading of 10 molar % free HPPH into liposomes. Liposomes containing 50 mol. % DSPC, 35 mol. % cholesterol, 5 mol. % DSPE-PEG2K and 10% free HPPH were formed from the thin film method, hydrated with buffered saline, sonicated and subjected to gel filtration. The elution fractions of liposomes and free molecules are indicated. All samples were lysed with 0.25% Triton-X100 prior to fluorescence measurement of HPPH.

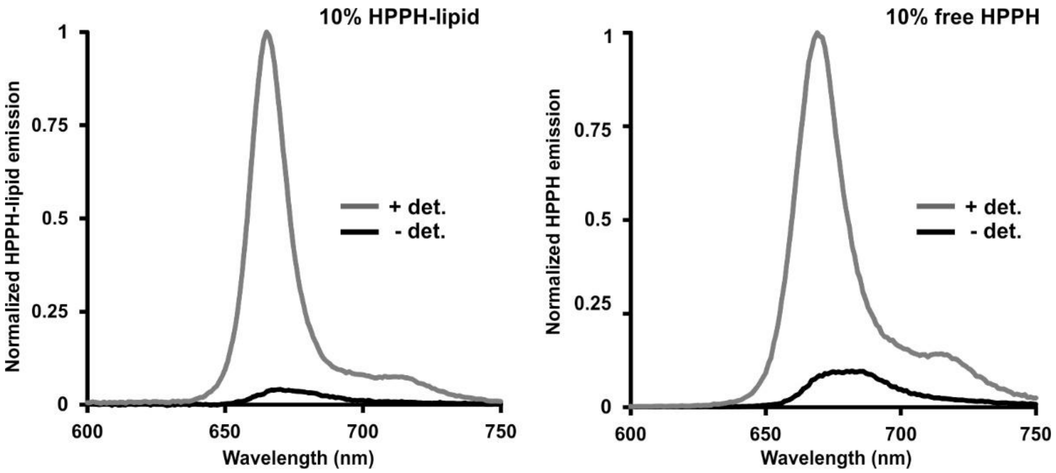

Supplementary Figure 3: Fluorescence self-quenching of PoP-liposomes containing 10 molar % HPPH-lipid or 10 molar % free HPPH. Liposomes were formed with 50 mol. % DSPC, 35 mol. % cholesterol, 5 mol. % DSPE-PEG2K, and 10 mol. % either free HPPH or HPPH-lipid. Liposomes formed with 10% HPPH-lipid exhibited HPPH fluorescence quenching of 97% (~30 fold) whereas liposomes containing 10% free HPPH were quenched 92% (~13 fold). Samples were measured in PBS and 0.25% Triton-X100 was used to lyse the liposomes.

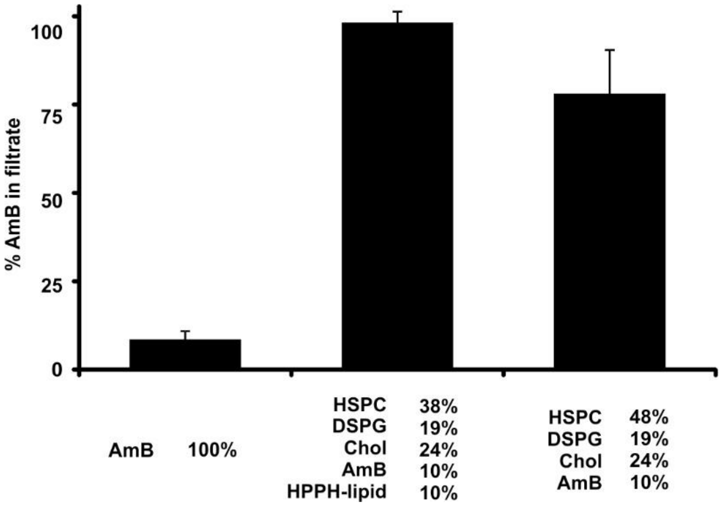

Supplementary Figure 4: HPPH-lipid does not interfere with Amphotericin B loading in membranes. Amphotericin B (AmB) was loaded into liposomes composed of the indicated lipids or free amphotericin B via thin film hydration of 10 mg films. The samples were sonicated and filtered and the resulting amphotericin B in percentage of soluble AmB that could enter the filtrate was assessed via fluorescence (mean +/- std. dev. for n=3).

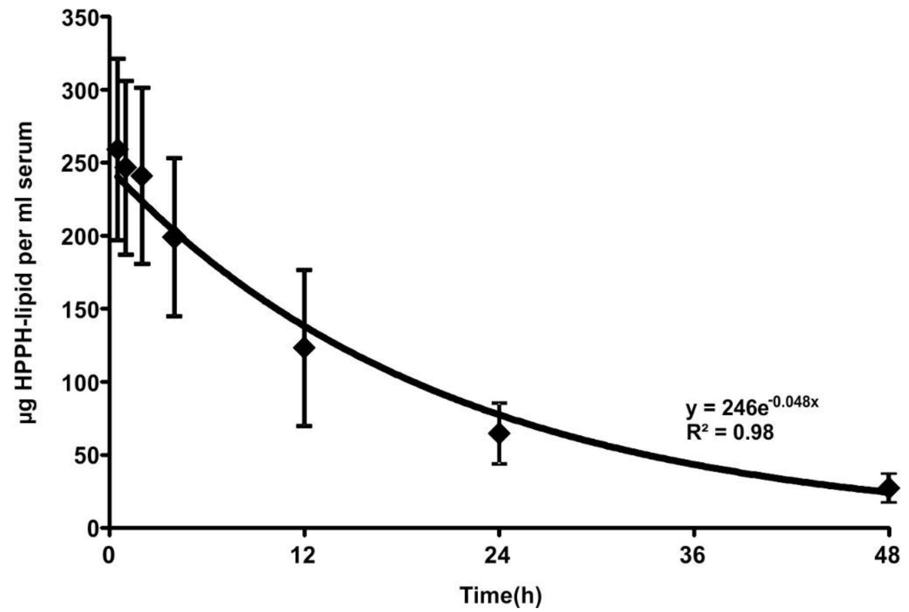

Supplementary Figure 5: Half-life of PoP-liposomes in mice following intravenous injection. PoP-liposomes (composed of 50 mol. % DPSC, 35 mol. % cholesterol, 10 mol. % HPPH-lipid and 5 mol. % DSPE-PEG2K) were injected via tail vein into BALB/c mice (15 mg/kg based on HPPH-lipid). Serum was sampled and HPPH-lipid was detected using fluorescence. A single compartment circulating half-life of 14.4 hrs was observed (std. dev. of 1.4 hours for n = 5 mice).

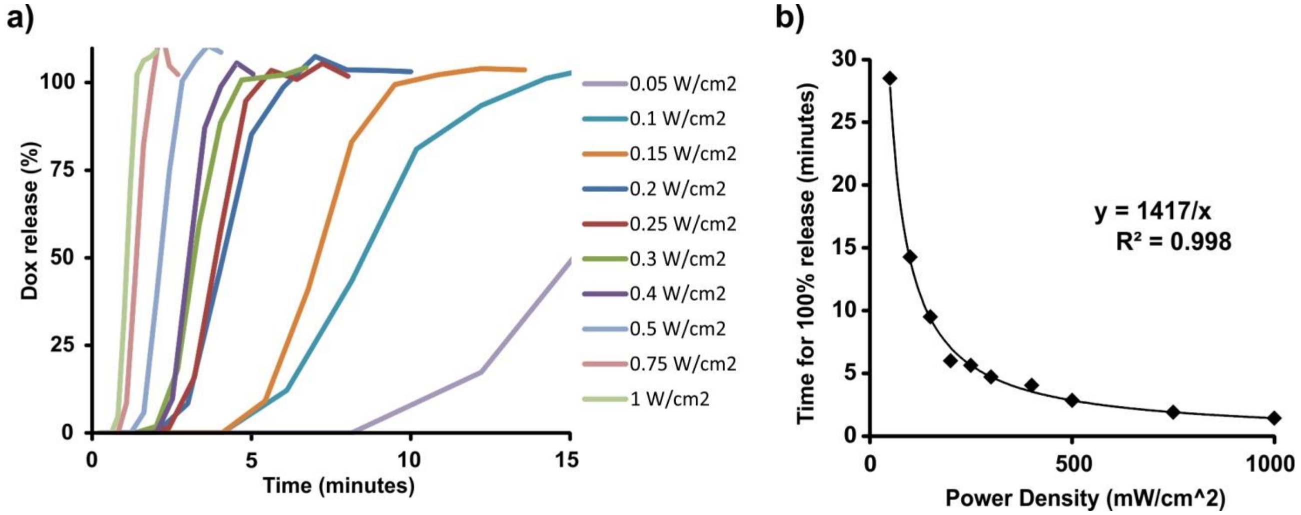

Supplementary Figure 6: Release of doxorubicin depends only on total fluence, regardless of fluence rate. a) Doxorubicin release from PoP-liposomes as a function of time. **b)** Time required for 100% doxorubicin release at different power densities.

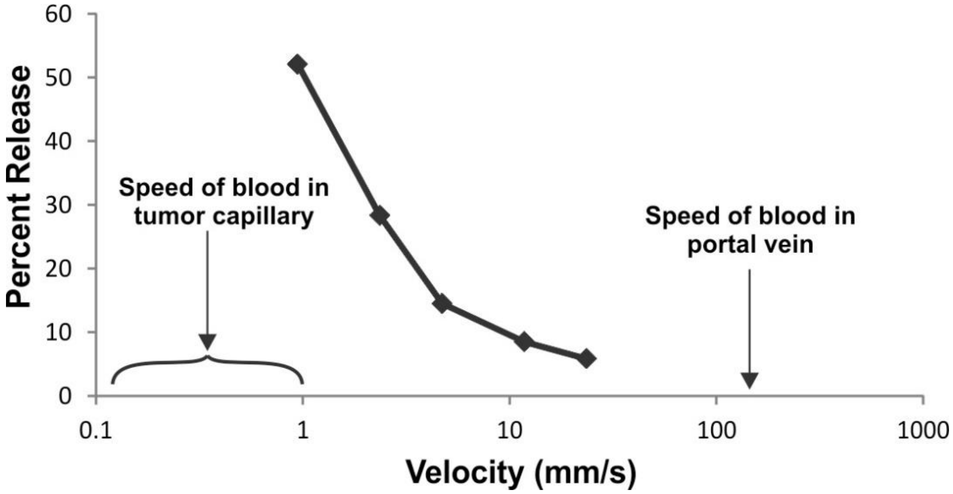

Supplementary Figure 7: Flow rates affects cargo release from PoP-liposomes. Calcein loaded PoP-NVs were moved through capillary tubing at the indicated velocities under irradiation and release was measured following fluid exit from the tubing. For reference, speed of blood in tumor capillaries and major vessels (e.g. portal vein) is shown.

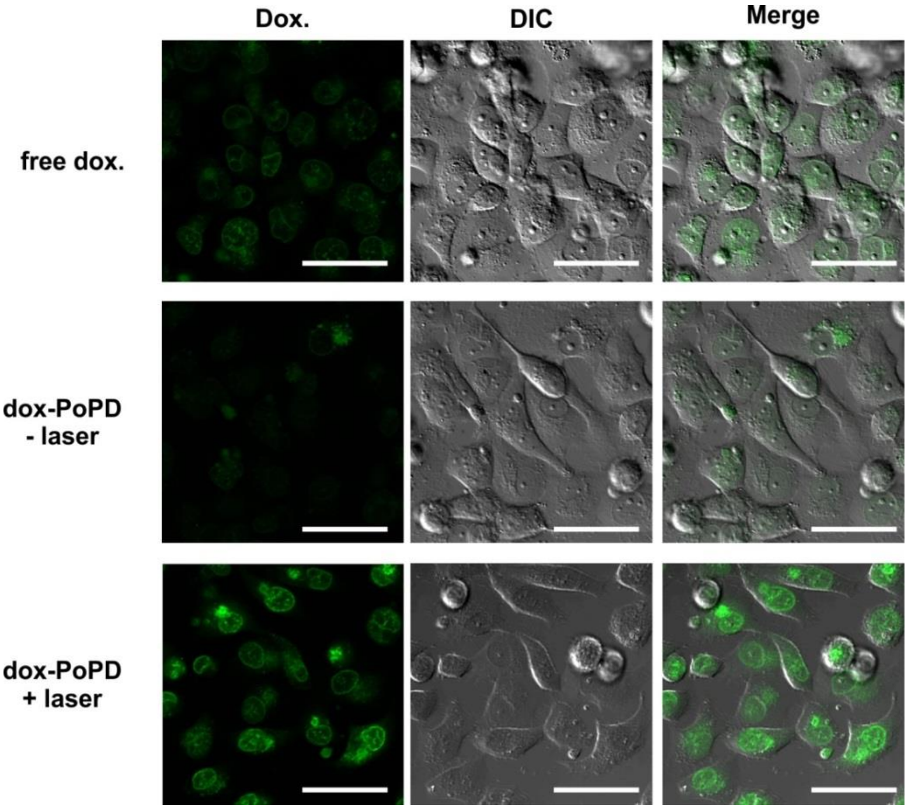

Supplementary Figure 8: Doxorubicin release from PoP-liposomes in Panc-1 cells. Panc-1 cells were seeded in chamber slides and incubated for 3 hours in 10% serum with 10 µg/mL doxorubicin in either free or PoP-liposomal form prior to confocal imaging. Laser treatment occurred at the beginning of the incubation period (658 nm, 200 mW/cm^2, 5 minute treatment). 50 µm scale bar is shown.

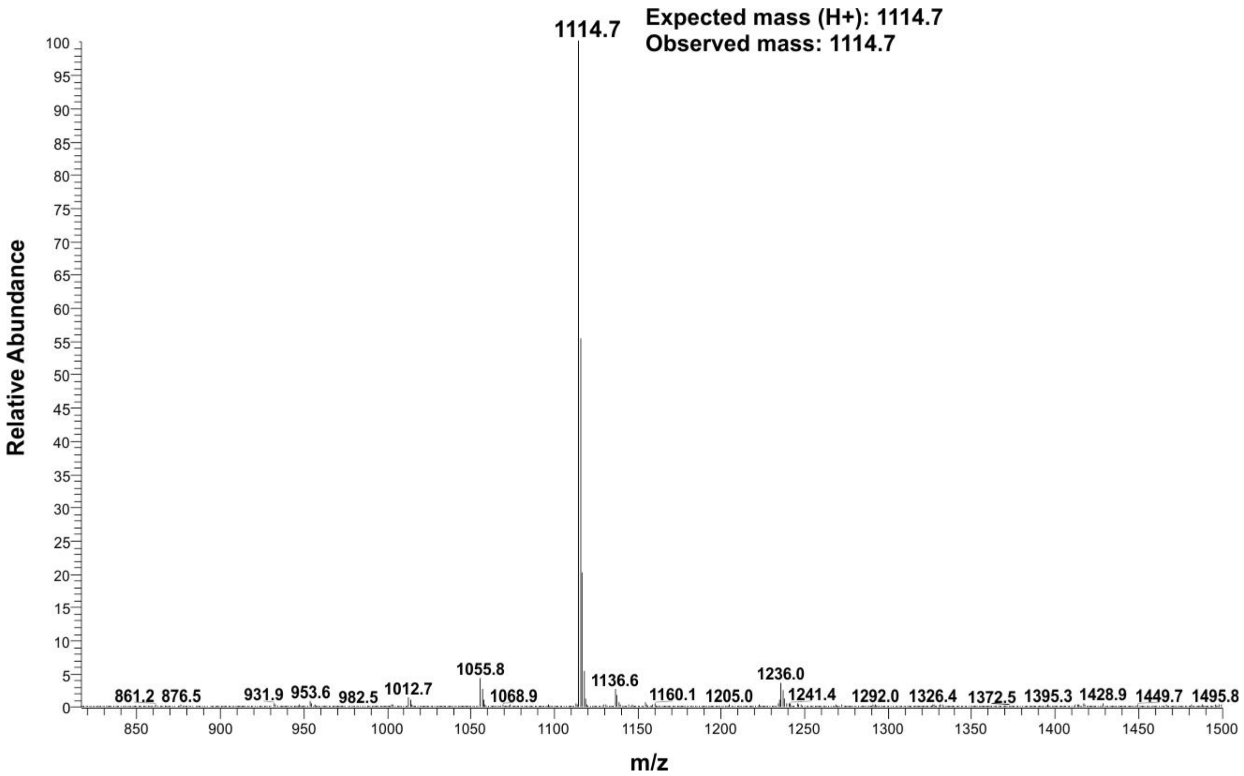

Supplementary Figure 9: Mass spectrum of HPPH-lipid.

Supplementary Note 1: NMR of HPPH-lipid

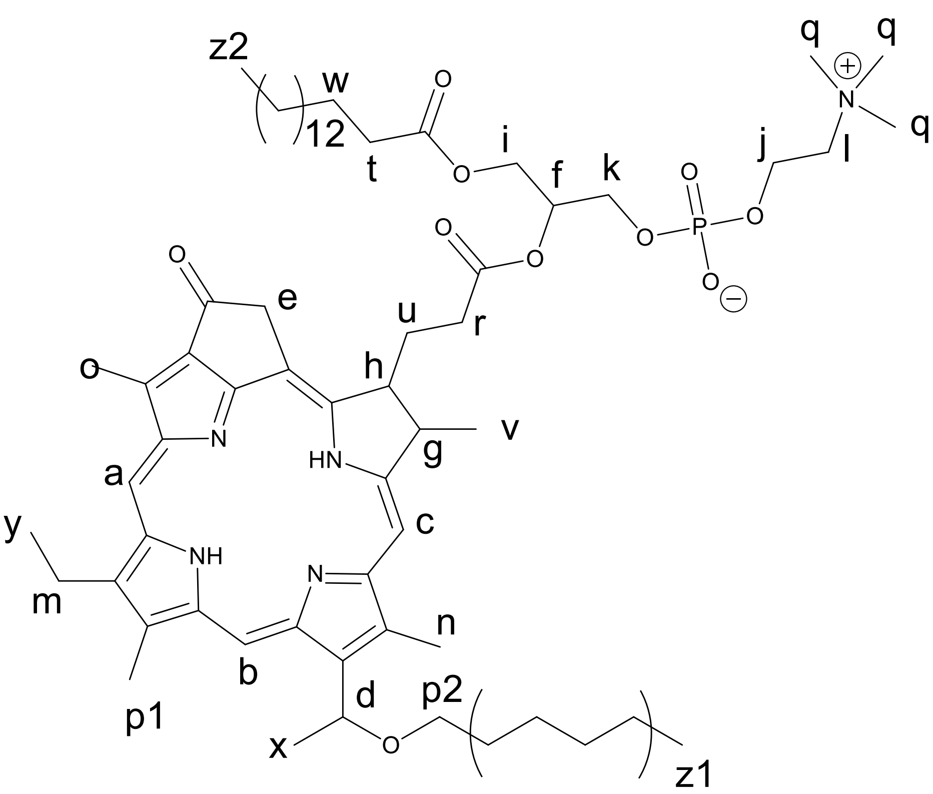

^{1}H NMR (500 MHz, $CDCl_3$) δ 9.79(a) (d, *J* = 11.2 Hz, 1H, meso-H), 9.40(b) (s, 1H, meso-H), 8.54(c) (d, *J* = 8.8 Hz, 1H, meso-H), 5.92 – 5.85(d) (m, 1H, CH(o-hexyl)CH_3), 5.36 – 5.20(e) (m, 2H, exocyclic ring), 5.13(f) (dd, *J* = 19.7 Hz, 1H, CH(CH_2)$_2$), 4.50(g) and 4.30(i) (d, *J* = 6.7 Hz, 2H, $COOCH_2CH$), 4.39(h) (s, 1H, CH(CH)CH_2), 4.30(j) (s, 1H, CH(CH)CH_2), 4.18-4.06(k) (t, J=12.8, 2H, OCH_2CH_2), 3.84(l) (d, *J* = 23.2 Hz, 2H, CH_2(CH)(PO_4)), 3.66(m) (d, *J* = 7.8 Hz, 2H, CH_2(CH_2)N(CH_3)$_3$), 3.57(n)(m, 2H, CH_2CH_3), 3.38(o) (s, 3H, C-CH_3), 3.33(p) (s, 3H, C-CH_3), 3.27(q1) (S, 3H, C-CH_3), 3,27(q2) (t, J=11.6, 2H, OCH_2), 3.08(r) (s, 9H, N(CH_3)$_3$), 2.75(s) (m, 2H, CH_2COO), 2.26-2.16(t) (t, J=14.7, 2H, CH_2(CH_2)COO), 2.11(u) (d, *J* = 6.4 Hz, 2H, CH_2(CH)CH_2COO), 1.81(v) (dd, *J* = 4.8 Hz, 2H, CH_2(CH_2)(CH_2)$_{12}$), 1.73(w) (d, *J* = 22.4 Hz, 3H, CH_3CH), 1.68(x) (t, *J* = 7.5 Hz, 3H, CH_3CH_2), 1.49(y) (d, *J* = 7.3 Hz, 3H, CH_3CH), 1.36 – 0.95(z) (several H, (CH_2)$_{12}$ and (CH_2)$_4$), 0.86(a1) (t, *J* = 6.9 Hz, 3H, CH_3CH_2), 0.79(b1) (s, 3H, CH_3CH_2), -1.74 (br, 1H).
